# Penalized Variable Selection for Joint AFT Random‐Effect Model With Clustered Competing‐Risks Data

**DOI:** 10.1002/pst.70084

**Published:** 2026-03-15

**Authors:** Lin Hao, Il Do Ha

**Affiliations:** ^1^ College of Economics Management Weifang University of Science and Technology Shouguang China; ^2^ Department of Statistics and Data Science Pukyong National University Busan Korea

**Keywords:** AFT random‐effect model, clustered competing risks data, competing risks models, H‐likelihood, penalized variable selection

## Abstract

Clustered competing‐risks data often arise in clinical studies, such as multi‐center clinical trials, where the occurrence of an event within a cluster hinders the observation of other types of events. The correlation resulting from clustering can be modeled using random effects. These competing‐risks data have usually been analyzed using hazard‐based models, rather than survival times themselves. Hao et al. proposed a cause‐specific joint accelerated failure time (AFT) random‐effect modeling approach for analyzing the clustered competing‐risks data, which is easy to interpret. In this article, we propose a variable selection method for fixed effects using a penalized h‐likelihood (HL) procedure in the joint AFT competing‐risk model. Simulation studies were conducted to evaluate the performance of the proposed variable selection procedure, which concluded that the penalized methods of SCAD and HL are more appropriate than that of LASSO. The proposed method is illustrated with two real clinical datasets.

## 1 Introduction

Clustered competing‐risks data [1] are often encountered in the biomedical field including multi‐center clinical trials, where the occurrence of an event within a cluster hinders the observation of other types of events. These clustered data which are correlated within a cluster can be modeled via random effects. For the analysis of competing risks data, three different types of hazard functions have been considered, that is, the cause‐specific hazard, the sub‐distribution hazard (subhazard), and the marginal hazard function. All three classes of hazard models have been extended to clustered competing risks data by incorporating frailty terms [2, 3, 4, 5]. Very recently, Hao et al. [6] proposed a cause‐specific joint parametric AFT random‐effect model for clustered competing‐risks data which is easy to interpret. In particular, the estimated regression parameters in the AFT models are robust against violation of the model assumption [7, 8]. In this paper we are interested in variable selection of fixed effects (regression parameters) in the cause‐specific AFT random‐effect model by Hao et al. [6].

Penalized variable selection approaches with various penalty functions based on the likelihood have been widely studied in various statistical models such as the Cox proportional hazards (PH) models and generalized linear models (GLMs) [2]. The main advantage of these variable selection approaches is to select important covariates and to estimate fixed effects simultaneously; it deletes insignificant variables by estimating their effects as zero [9].

Park and Ha [10] proposed a variable selection procedure of fixed effects in an AFT random‐effect model for clustered survival data without competing risks, using penalized h‐likelihood [11] approaches. The h‐likelihood avoids the difficult integration with respect to the random effects required to obtain marginal likelihood [12, 13]. Thus, in this paper, we propose a penalized h‐likelihood method for variable selection of fixed effects in the cause‐specific joint AFT model [6], which is an extension of Park and Ha's [10] method to the clustered competing‐risk setting. Here, we study three penalty functions: LASSO (least absolute shrinkage and selection operator [14]), SCAD (smoothly clipped absolute deviation [9]), and HL (h‐likelihood penalty [11]).

The rest of this article is organized as follows. In Section 2, we review the cause‐specific AFT random‐effect model for clustered competing‐risks data, and outline the corresponding h‐likelihood. In Section 3, a variable selection procedure for the cause‐specific AFT random‐effect model is proposed using a penalized h‐likelihood method. Simulation studies for evaluating the performance of the proposed method are presented in Section 4. Section 5 illustrates the proposed method with two real‐data sets. In Section 6, we conclude with a discussion.

## 2 Joint Aft Competing‐Risks Model and H‐Likelihood

In this section, we briefly describe the joint AFT competing‐risks model allowing for random effect which was proposed by Hao et al. [6], and outline the h‐likelihood construction for the model estimation.

### 2.1 The Model

For the *j*‐th $\left(j=1,\ldots ,n_{i}\right)$ subject in the *i*‐th $\left(i=1,\ldots ,q\right)$ cluster (center), let $T_{\mathrm{ijk}}$ be the Type k event time from cause $k\;\left(k=1,\ldots ,K\right)$. In this paper, for simplicity, we consider two types of events $\left(K=2\right)$. Specifically, it indicates the event of interest (main event) from cause 1 when k=1, whereas it is the competing event from cause 2 when k=2. Here, n is the number of clusters and $n_{i}$ is cluster size (i.e., the number of observations (subjects) in the *i*‐th cluster); thus, the total sample size is $n=\sum_{i=1}^{q}n_{i}$. Denote $T_{\mathrm{ij}}=\min\left(T_{\mathrm{ij}1},T_{\mathrm{ij}2}\right)$ as the time to the first event, and denote $\xi_{\mathrm{ij}}\in \left\{1,2\right\}$ as the corresponding cause of event (type of event). Let $C_{\mathrm{ij}}$ be the independent censoring time. Let $V_{i}\;\left(i=1,\ldots ,q\right)$ be an unobserved shared random effect of the *i*‐th cluster. Assume that given $V_{i}=v_{i}$, $C_{\mathrm{ij}}$ is conditionally independent and non‐informative of ($T_{\mathrm{ij}},\xi_{\mathrm{ij}}$) for $j=1,\ldots ,n_{i}$ [2, 6, 15].

The cause‐specific joint AFT model with a shared random effect for clustered competing‐risks data, proposed by Hao et al. [6], is described as follows:

(1) $$\begin{array}{l} \left(i\right)\;\log T_{\mathrm{ij}1}=x_{\mathrm{ij}}^{T}\beta_{1}+V_{i}+\epsilon_{\mathrm{ij}1},\\ \left(\mathrm{ii}\right)\;\log T_{\mathrm{ij}2}=x_{\mathrm{ij}}^{T}\beta_{2}+\gamma V_{i}+\epsilon_{\mathrm{ij}2}, \end{array}$$

where $\beta_{k}={\left(\beta_{k0},\beta_{k1},\ldots ,\beta_{k,p-1}\right)}^{T}\;\left(k=1,2\right)$ are p×1 vectors of fixed effects (regression parameters) for Type $k\;\left(k=1,2\right)$ events, and $x_{\mathrm{ij}}={\left(1,x_{\mathrm{ij}1},\ldots ,x_{\mathrm{ij},p-1}\right)}^{T}$ is a p×1 vector of fixed covariates corresponding to $\beta_{k}$. Here, $V_{i}\overset{\mathrm{iid}}{∼}N\left(0,\alpha \right)$ is an unobserved shared random effect for the *i*‐th cluster, $\epsilon_{\mathrm{ijk}}∼N\left(0,\phi_{k}\right)\;\left(k=1,2\right)$ are independent random error terms, and $V_{i}$ and $\epsilon_{\mathrm{ijk}}\;\left(k=1,2\right)$ are also independent. Note that γ is a real‐valued association parameter that accounts for the dependency between Type 1 and Type 2 events. In particular, γ has similar behavior (e.g., the same sign) to correlation coefficient, $\rho =\text{corr}\left(\log T_{\mathrm{ij}1},\log T_{\mathrm{ij}2}\right)$, between $\log T_{\mathrm{ij}1}$ and $\log T_{\mathrm{ij}2}$ [6]. The model (1) is a fully parametric, but the estimated regression parameters are robust against violation of the model assumption [6]. The interpretation of model (1) is easy because fixed and random effects act linearly on each individual's survival time [2, 7]. For example, in the Type 1 case with a single binary covariate x (x=1 for new drug and x=0 for placebo), the survival time $T_{1}$ in the new drug group is increased by a factor of $\exp\left(\beta_{1}\right)$ compared to the placebo if the corresponding regression coefficient $\beta_{1}$ is positive.

### 2.2 H‐Likelihood Construction

The observed event times and event indicator are, respectively, defined by:

(2) $$Y_{\mathrm{ij}}=\min\left(\log T_{\mathrm{ij}},\log C_{\mathrm{ij}}\right)\;\text{and}\;\delta_{\mathrm{ijk}}=I\left(Y_{\mathrm{ij}}=\log T_{\mathrm{ijk}}\right),$$

where $\delta_{\mathrm{ijk}}$ is the censoring indicator of the *k*‐th $\left(k=1,2\right)$ type of event, which means that $\delta_{\mathrm{ijk}}=1$ if Type k event occurs first ($Y_{\mathrm{ij}}=\log T_{\mathrm{ijk}}$) and 0 otherwise. Here, $I\left(\cdot \right)$ is the indicator function.

Following previous literature [2, 6, 15], the h‐likelihood for the joint AFT random‐effect model (1) is defined as:

(3) $$h=h\left(\beta_{1},\beta_{2},v,\phi_{1},\phi_{2},\alpha ,\gamma \right)=\sum_{k=1}^{2}\left(\sum_{\mathrm{ij}}\ell_{1\mathrm{ijk}}\right)+\sum_{i}\ell_{2i},$$

where

(4) $$\ell_{1\mathrm{ijk}}=-\delta_{\mathrm{ijk}}\left(\log\left(2{\pi \phi }_{k}\right)+m_{\mathrm{ijk}}^{2}\right)/2+\left(1-\delta_{\mathrm{ijk}}\right)\log\left\{1-\Phi \left(m_{\mathrm{ijk}}\right)\right\},k=1,2,$$

are the conditional log‐likelihoods for ($Y_{\mathrm{ij}},\delta_{\mathrm{ijk}}$) (k=1,2) given $V_{i}=v_{i}$, respectively, and $\Phi \left(\cdot \right)$ is the cumulative distribution function of $N\left(0,1\right)$. Note here that $m_{\mathrm{ijk}}=\left(y_{\mathrm{ij}}-\mu_{\mathrm{ijk}}\right)/\sqrt{\phi_{k}}\;\left(k=1,2\right)$, where $\mu_{\mathrm{ij}1}=E\left(\log T_{\mathrm{ij}1}|V_{i}=v_{i}\right)=x_{\mathrm{ij}}^{T}\beta_{1}+v_{i}$, $\mu_{\mathrm{ij}2}=E\left(\log T_{\mathrm{ij}2}|V_{i}=v_{i}\right)$ $=x_{\mathrm{ij}}^{T}\beta_{2}+\gamma v_{i}$, and

(5) $$\ell_{2i}=-\log\left(2\pi \alpha \right)/2-v_{i}^{2}/\left(2\alpha \right),$$

is the log‐likelihood for $V_{i}$.

Then, the h‐likelihood (3) can be expressed as

(6) $$\begin{array}{l} h=\sum_{\mathrm{ijk}}\left\{-\frac{1}{2}\delta_{\mathrm{ijk}}\left(\log\left(2{\pi \phi }_{k}\right)+m_{\mathrm{ijk}}^{2}\right)+\left(1-\delta_{\mathrm{ijk}}\right)\log\left\{1-\Phi \left(m_{\mathrm{ijk}}\right)\right\}\right\}\\ -\frac{1}{2}\sum_{i}\left\{\log\left(2\pi \alpha \right)+\frac{v_{i}^{2}}{\alpha }\right\}. \end{array}$$

Accordingly, hereafter the h‐likelihood inference is based on (6) [2, 6].

## 3 Variable Selection Procedure

Ha et al. [16] and Park and Ha [10] proposed a penalized variable selection procedure for fixed effects in the standard frailty model and AFT random‐effect model, respectively. Furthermore, Rakhmawati et al. [17] and Ha et al. [3] proposed the penalized h‐likelihood methods in cause‐specific and subdistribution hazard competing‐risks frailty models, respectively. For the variable selection of regression parameters β in the cause‐specific joint AFT random‐effect model (1), following Ha et al. [10, 16], we propose a penalized h‐likelihood $h_{p}$, using h in (6) and a penalty; it is defined by

(7) $$h_{p}\left(\beta ,v,\theta \right)=h-n\sum_{j^{*}=1}^{p^{*}}J_{\lambda }\left(\left|\beta_{j^{*}}\right|\right),$$

where $\beta ={\left(\beta_{1}^{T},\beta_{2}^{T}\right)}^{T}$, $p^{*}=\mathrm{Kp}$ with K=2, and $J_{\lambda }\left(|\cdot |\right)$ is a penalty term that is a function of the tuning parameter λ to control model complexity. Note here that the intercepts are being penalized, even though this is not common in practice. A larger value of λ tends to choose a simpler model, whereas a smaller value of λ leads to a more complex model. Note that the penalty term is related only to the fixed effects β and has nothing to do with the dispersion parameters $\theta ={\left(\phi_{1},\phi_{2},\alpha ,\gamma \right)}^{T}$. A type of Bayesian information criterion (BIC) [10, 16], described in (15) of Section 3.2, can be used for the selection of the optimal tuning parameter. The tuning parameter cannot be adequately selected using generalized cross‐validation due to the inevitable overfitting issue [18, 19].

**FIGURE 1:**
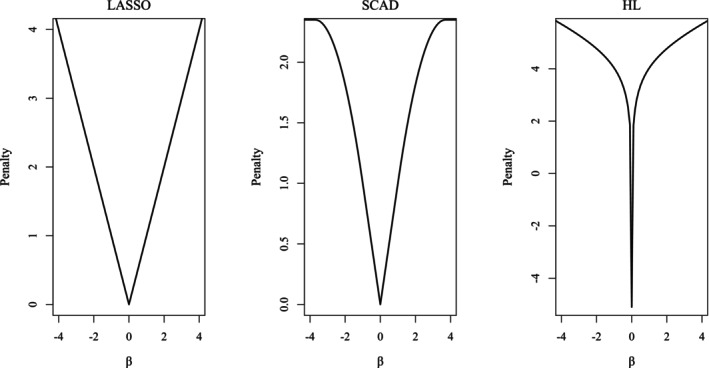
Plots of three penalty functions.

In this paper, we study three penalty functions, as shown in Figure 1; however, our methodology can be applied to other penalty functions beyond these three.

- LASSO [14]

$$J_{\lambda }\left(|\beta |\right)=\lambda |\beta |.$$

ii SCAD [9]
$$J_{\lambda }^{\prime }\left(|\beta |\right)=\mathrm{\lambda I}\left(|\beta |\leq \lambda \right)+\frac{{\left(\mathrm{a\lambda}-|\beta |\right)}_{+}}{a-1}I\left(|\beta |>\lambda \right),$$
  where a=3.7, and ${\left(\cdot \right)}_{+}$ indicates the positive part of $\left(\cdot \right)$.
iii HL [11]

$$\begin{array}{c} J_{\lambda }\left(|\beta |\right)=J_{\left(\lambda ,b\right)}\left(|\beta |\right)=\log\Gamma \left(\frac{1}{b}\right)+\frac{\log b}{b}+\frac{\beta^{2}}{2\mathrm{\lambda u}\left(|\beta |\right)}\\ \;+\frac{\left(b-2\right)\log u\left(|\beta |\right)}{2b}+\frac{u\left(|\beta |\right)}{b} \end{array}$$

where

$$u\left(|\beta |\right)=\left[{\left\{8b\beta^{2}/\lambda +{\left(2-b\right)}^{2}\right\}}^{1/2}+2-b\right]/4.$$

It is well known that a good penalty function should produce estimates that satisfy the three oracle properties, that is, unbiasedness, sparsity, and continuity [9, 20]. Of the three penalty functions above, LASSO is the most common penalty. However, it does not meet the three oracle properties, and it usually selects a model with too many variables to prevent over‐shrinkage of regression coefficients. In contrast, SCAD and HL meet the three oracle properties, and they have good performances in terms of estimating true nonzero coefficients and selecting correct subset models simultaneously [9, 11]. The shape of the HL penalty function changes according to the value of b. When b is 0, it becomes a ridge penalty; when b is 2, it becomes a LASSO penalty; and it becomes an unbounded form at the origin when b>2 [11], which means a singular unbounded penalty at zero with $J_{\lambda }\left(0\right)=-\infty$. As the convexity near the origin increases, the sparsity of the local solutions increases, and as the slope becomes flat, the amount of shrinkage lessens. We thus see that the HL controls the sparsity and shrinkage amount simultaneously [11, 13]. Following Lee and Oh [11], in this paper, we use b=30 [17] for the HL penalty as shown in Figure 1.

### 3.1 Estimation Procedure for Variable Selection

For the estimation of fixed and random effects $\tau ={\left(\beta^{T},v^{T}\right)}^{T}$, the penalized maximum h‐likelihood (PMHL) estimators which maximize $h_{p}$ in (7) are applied. Given dispersion parameters $\theta ={\left(\phi_{1},\phi_{2},\alpha ,\gamma \right)}^{T}$, the PMHL estimating equations of $\beta ={\left(\beta_{1}^{T},\beta_{2}^{T}\right)}^{T}$ and v are, respectively, given by

(8) $$\begin{array}{l} \frac{\partial h_{p}}{\partial \beta }=\frac{\partial h}{\partial \beta }-n\sum_{j^{*}=1}^{p^{*}}{\left(J_{\lambda }\left(\left|\beta_{j^{*}}\right|\right)\right)}^{\prime }=0,\\ \frac{\partial h_{p}}{\partial v}=\frac{\partial h}{\partial v}=0. \end{array}$$

The first equation in (8) includes a penalty term, yet the second equation in (8) (i.e., the PMHL estimating equation for the random effect v) is the same as that without penalty [10]. Following Ha et al. [7] and Hao et al. [6], $\partial h/\partial \beta_{k}\left(k=1,2\right)$ and ∂h/∂v are given as follows:
(9)

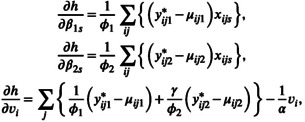

where $y_{\mathrm{ijk}}^{*}=\delta_{\mathrm{ijk}}y_{\mathrm{ij}}+\left(1-\delta_{\mathrm{ijk}}\right)\left\{\mu_{\mathrm{ijk}}+\sqrt{\phi_{k}}U\left(m_{\mathrm{ijk}}\right)\right\}\;\left(k=1,2\right)$ and $\mu_{\mathrm{ijk}}\;\left(k=1,2\right)$ are given in (4).

Note that, it is difficult to solve the PMHL estimating equation of β in (8) directly as the penalty term $J_{\lambda }\left(|\beta_{j^{*}}|\right)$ becomes a non‐differentiable function at the origin and it also does not have the continuous second‐order derivative. Hence, to overcome this difficulty, a local quadratic approximation (LQA) [9] method is used for such penalty terms. Given an initial value $\beta^{\left(0\right)}$ that is close to the true value of β, the penalty function $J_{\lambda }\left(|\cdot |\right)$ can be approximated locally by a quadratic function, which is given by

(10) $${\left(J_{\lambda }\left(\left|\beta_{j^{*}}\right|\right)\right)}^{\prime }=J_{\lambda }^{\prime }\left(\left|\beta_{j^{*}}\right|\right)\;\mathrm{sgn}\;\left(\beta_{j^{*}}\right)\approx \left\{J_{\lambda }^{\prime }\left(\left|\beta_{j^{*}}^{\left(0\right)}\right|\right)/\left|\beta_{j^{*}}^{\left(0\right)}\right|\right\}\cdot \beta_{j^{*}}\;\text{for}\;\beta_{j^{*}}\approx \beta_{j^{*}}^{\left(0\right)}.$$

In order to obtain the penalized estimating equations of $\tau ={\left(\beta^{T},v^{T}\right)}^{T}$, we define the following partition matrices $X^{*}=\left(\begin{array}{cc} X & 0\\ 0 & X \end{array}\right)$, $W^{*}=\left(\begin{array}{cc} W_{1}^{*} & 0\\ 0 & W_{2}^{*} \end{array}\right)$ and $Z^{*}=\left(\begin{array}{c} Z\\ \mathrm{\gamma Z} \end{array}\right)$.

In which, $W_{k}^{*}=W_{k}/\phi_{k}\;\left(k=1,2\right)$ with $W_{k}$ being diagonal matrices with elements $w_{\mathrm{ijk}}$. Here, $w_{\mathrm{ijk}}=\delta_{\mathrm{ijk}}+\left(1-\delta_{\mathrm{ijk}}\right)\zeta \left(m_{\mathrm{ijk}}\right)\left(k=1,2\right)$ with $\zeta \left(m_{\mathrm{ijk}}\right)=\partial U\left(m_{\mathrm{ijk}}\right)/\partial m_{\mathrm{ijk}}=U\left(m_{\mathrm{ijk}}\right)\left(U\left(m_{\mathrm{ijk}}\right)-m_{\mathrm{ijk}}\right)$ and $U\left(\cdot \right)=\varphi \left(\cdot \right)/\left(1-\Phi \left(\cdot \right)\right)$ is the hazard function of $N\left(0,1\right)$. For details, see Hao et al. [6] and Park and Ha [10]. X and Z are model matrices of fixed effects $\beta_{k}\;\left(k=1,2\right)$ and random effects v, respectively. According to Hao et al. [6] and Park and Ha [10], we can show that the PMHL estimators for $\tau ={\left(\beta^{T},v^{T}\right)}^{T}$ can be obtained by the following IWLS score equations with a penalty term:

(11) $$\left(\begin{array}{cc} X^{*T}W^{*}X^{*}+n\Sigma_{\lambda } & X^{*T}W^{*}Z^{*}\\ Z^{*T}W^{*}X^{*} & Z^{*T}W^{*}Z^{*}+Q \end{array}\right)\left(\begin{array}{l} \hat{\beta }\\ \hat{v} \end{array}\right)=\left(\begin{array}{c} X^{*T}w^{**}\\ Z^{*T}w^{**} \end{array}\right),$$

where $\sum_{\lambda }$ is a $p^{*}\times p^{*}$ diagonal matrix with elements $J_{\lambda }^{\prime }\left(|\beta_{j^{*}}|\right)/|\beta_{j^{*}}|$. $\hat{\beta }={\left({{\hat{\beta }}_{1}}^{T},{{\hat{\beta }}_{2}}^{T}\right)}^{T}$ and $w^{**}={\left({w_{1}^{**}}^{T},{w_{2}^{**}}^{T}\right)}^{T}$ with ${w_{k}^{**}}^{T}=W_{k}^{*}\mu_{k}+\left(y_{k}^{*}-\mu_{k}\right)/\phi_{k}\left(k=1,2\right)$ being $n^{*}\times 1$ vectors; $n^{*}=\mathrm{Kn}$ and $p^{*}=\mathrm{Kp}$ with K=2. Here, $\mu_{k}$ and $y_{k}^{*}$ are n×1 vectors of $\mu_{\mathrm{ijk}}$ and $y_{\mathrm{ijk}}^{*}\;\left(k=1,2\right)$, respectively. $Q=I_{q^{*}/\alpha }$ with $I_{q^{*}}$ being the $q^{*}\times q^{*}$ identity matrix. Note that the score Equations (11) are the extensions of the existing estimation procedures. For example, under no penalty (i.e., $\sum_{\lambda }=0$) they become the score equations of Hao et al. [6] for the standard cause‐specific joint AFT model.

Moreover, the dispersion parameters $\theta ={\left(\phi_{1},\phi_{2},\alpha ,\gamma \right)}^{T}$ are estimated by maximizing the adjusted profile h‐likelihood $p_{\tau }\left(h\right)$ [6, 10] as dispersion parameters θ are not related to the penalty term in (7); it is given by a function of θ, that is,

(12) $$p_{\tau }\left(h\right)={\left.\left[h-\frac{1}{2}\log\det\left(H\right)+\frac{2\left(p+q\right)}{2}\log\left(2\pi \right)\right]\right|}_{\tau =\hat{\tau }},$$

where $H=H\left(h;\tau \right)=-\partial^{2}h/\partial \tau \partial \tau^{T}$ is given by the square matrix of the left‐hand corner of (11). Here, $\tau ={\left(\beta^{T},v^{T}\right)}^{T}$ and $\hat{\tau }=\hat{\tau }\left(\theta \right)={\left(\hat{\beta }{\left(\theta \right)}^{T},\hat{v}{\left(\theta \right)}^{T}\right)}^{T}$. Then, the estimates of θ are obtained by solving the following score equations:

(13) $$\frac{\partial p_{\tau }\left(h\right)}{\partial \theta }=0.$$

It can be seen that the proposed variable selection procedure for the model (1) is easily implemented by slightly modifying the penalized h‐likelihood procedures for the AFT random‐effect model [10] and cause‐specific hazard frailty model [17].

### 3.2 Standard Error and Selection of Tuning Parameter

Following Ha et al. [10, 16] and Rakhmawati et al. [17], approximated standard errors (SEs) of β can be obtained by a sandwich formula based on $h_{p}$ in (7):

(14) $$\mathrm{cov}\left(\hat{\beta }\right)={\left(H_{\beta \beta }+n\Sigma_{\lambda }\right)}^{-1}H_{\beta \beta }{\left(H_{\beta \beta }+n\Sigma_{\lambda }\right)}^{-1},$$

where $\begin{array}{c} H_{\beta \beta }=\left.\left\{\left(X{*}^{T}W*X*\right)-\left(X{*}^{T}W*Z*\right){\left(Z{*}^{T}W*Z*+Q\right)}^{-1}\right.\right. \end{array}$ $\begin{array}{c} {\left.\left.\left(Z{*}^{T}W*X*\right)\right\}\right|}_{v=\hat{v}} \end{array}$. A BIC‐type criterion based on the penalized h‐likelihood [10, 17] is applied for the selection of tuning parameter λ, defined as

(15) $$\mathrm{BIC}\left(\lambda \right)=-2p_{v}\left(h\right)+e\left(\lambda \right)\log\left(n\right),$$

where $p_{v}\left(h\right)={\left.\left[h-\log\det\left(H_{v}/\left(2\pi \right)\right)/2\right]\right|}_{v=\hat{v}}$ is the first‐order Laplace approximation to the marginal likelihood $m\left(\beta ,\theta \right)=\log\left\{\int \exp\left(h\right)\mathrm{dv}\right\}$ [2, 16] with $H_{v}=-\partial^{2}h/\partial v\partial v^{T}$. Here, $e\left(\lambda \right)=\text{trace}\left({\left(H_{\beta \beta }+n\Sigma_{\lambda }\right)}^{-1}H_{\beta \beta }\right)$ is the effective number of parameters with $H_{\beta \beta }$ given in (14) [12, 13].

Below is the penalized variable selection algorithm for the model (1) which consists of two loops.

- **Step 0:** Determine the initial values of all parameters $\left(\tau ,\theta \right)$ for the three penalized variable selection methods (i.e., LASSO, SCAD, and HL). In the variable selection, a good initial value is also very important for obtaining a proper estimate $\hat{\beta }$. Thus, we take the solutions under no penalty (i.e., let $\sum_{\lambda }=0$ in (11)) as initial values for the LASSO penalty, and we take the LASSO solutions as initial values for both SCAD and HL penalties [10, 16].
- **Step 1 (inner loop):** Maximize $h_{p}$ (7) for $\tau ={\left(\beta^{T},v^{T}\right)}^{T}$ (i.e., solve the IWLS equations in (11) for $\left(\beta ,v\right)$ and $p_{\tau }\left(h\right)$ in (12) for θ, respectively).
- **Step 2 (outer loop):** Select the optimal λ (denoted by $\lambda^{*}$) that minimizes the $\mathrm{BIC}\left(\lambda \right)$ in (15), i.e., $\lambda^{*}=\underset{\lambda }{\arg\min}\;\mathrm{BIC}\left(\lambda \right)$, using a simple grid search method.

After convergence, we compute the estimated SEs for $\hat{\beta }$ using (14).

Furthermore, the SEs for the random effects v and dispersion parameters $\theta ={\left(\phi_{1},\phi_{2},\alpha ,\gamma \right)}^{T}$ are computed from the corresponding Hessian matrices as follows [2]. Let $H=H\left(h;\tau \right)=-\partial^{2}h/\partial \tau \partial \tau^{T}$ denote the square matrix of the left‐hand corner of (11). Then the approximated variance of $\hat{v}-v$ is obtained from the lower right‐hand corner of the inverse of H. Similarly, the variances of $\hat{\theta }$ are obtained from the inverse of $-\partial^{2}p_{\tau }\left(h\right)/\partial \theta \partial \theta^{T}$. Although they are not the primary focus of the current variable selection problem, these complex computations can be considered in future work, including the likelihood ratio tests on the random‐effect variance α and the association parameter γ.

## 4 Simulation Study

Referring to Fan and Li [20], numerical studies are conducted based on 100 replications of simulated data to evaluate the performance of the proposed variable‐selection procedure with three penalties (LASSO, SCAD, and HL) for the cause‐specific joint AFT random‐effect model (1).

### 4.1 Simulation Design

For these purposes above, we consider simulation schemes as follows:

- Sample sizes are $n=\sum_{i=1}^{q}n_{i}=200,400,600$, with $\left(n,n_{i}\right)=\left(20,10\right),\left(20,20\right),\left(20,30\right),\left(30,20\right)$, respectively.
- Generate covariates x by an AR(1) structure with a correlation coefficient ρ=0.5. That is, the covariate vector x is generated from a multivariate normal distribution with mean 0 and an AR(1) correlation matrix, that is, $\text{corr}\left(x_{i},x_{j}\right)=\rho^{|i-j|}$ with ρ=0.5; this induces stronger correlation among adjacent covariates, which decays exponentially with distance.
- Set the association parameter γ=−1. and 1.
- Set $\beta_{1}={\left(\beta_{10},\beta_{11},\ldots ,\beta_{18}\right)}^{T}={\left(0,0.8,0,0,1,0,0,0.6,0\right)}^{T}$ for both γ=1 and γ=−1.
- The true values of fixed effects in Type 2 event are set as $\beta_{2}=-\beta_{1}$ for both γ=−1 and γ=1.
- Set dispersion parameters α=0.5, and $\left(\phi_{1},\phi_{2}\right)=\left(3,1.5\right)$.
- The generations of survival time $T_{\mathrm{ij}}$ and censoring time $C_{\mathrm{ij}}$ are based on the design 1 in Hao et al. [6]. That is, the survival times $T_{\mathrm{ij}}$ are generated from the model (1) with two types using the inverse transformation method [17, 21, 22], and the survival times $C_{\mathrm{ij}}$ are generated from a uniform distribution. Here, under the censoring rate of 20%, the proportions of Type 1 [Type 2] event are about 24% [56%] or 40% [40%] corresponding to the case that the true value of the first component $\beta_{10}$ in $\beta_{1}$ is 1 or 0, respectively; for the 40% censoring, the corresponding proportions are about 17% [43%] and 30% [30%], respectively.

According to Fan and Li [20] and Ha et al. [16], the model error (ME) of the Type $k\;\left(k=1,2\right)$ event for the model (1) is defined by

(16) $$\mathrm{ME}\left({\hat{\beta }}_{k}\right)=E{\left(x^{T}{\hat{\beta }}_{k}-x^{T}\beta_{k}\right)}^{2}.$$

Below are criteria for analyzing the performances of three penalized variable selection methods (i.e., LASSO, SCAD, and HL).

- C: Average number of the true zero regression coefficients that are correctly set to zero under the Type 1 (or Type 2) event. Note that C =6 is the best.
- IC: Average number of true nonzero coefficients that are incorrectly found to be zero under the Type 1 (or Type 2) event. Obviously, IC =0 is the best.
- PT: Probability of choosing the true model under the Type 1 (or Type 2) event.
- MRME: Median of relative model error (RME), which is defined as the ratio of ME under the model with penalty (e.g., LASSO, SCAD, and HL) to that under the model without penalty (i.e., $\sum_{\lambda }=0$ in (11)). Note that MRME <1 is better.

Note that an R‐based program was developed to implement model fitting and computation.

### 4.2 Simulation Results

The simulation results under γ=1 are summarized in Table 1. From Table 1, SCAD and HL perform overall much better than LASSO in all sample sizes (i.e., N=200,400,600) in terms of “C”, “PT”, and “MRME”, regardless of whether the censoring rate is 20% or 40%. The performance of the other two methods (i.e., SCAD and HL) is generally much better as the sample size (n or $n_{i}$) increases. Supporting Information: Figures A.1 and A.2 present box plots in terms of, respectively, “MRME” and “C”, for three methods under both types of events when the censoring rate is 20%. Table 1 indicates that SCAD consistently outperforms HL in terms of “MRME”. However, the box plots in Supporting Information: Figures A.1 and A.2 show that there is no substantial difference between the SCAD and HL penalties. From Table 1; Supporting Information: Figures A.1 and A.2, we can see that HL overall outperforms SCAD in terms of “C” and “PT” when the censoring rate is 20%, which means that HL has a higher probability of choosing the true model than LASSO and SCAD. When the censoring rate is 40%, SCAD performs consistently better than LASSO and HL in terms of four criteria for the Type 1 event; while HL consistently outperforms SCAD in terms of “C” and “PT” for the Type 2 event.

**TABLE 1:** Simulation results using 100 replications for variable selection in the model (1) under γ=1.

Cen.	(q, $n_{i}$)	Method	Type 1				Type 2			
			C	IC	PT	MRME	C	IC	PT	MRME
20%	(10, 20)	LASSO	4.28	0.00	0.11	0.669	3.84	0.00	0.08	0.668
		SCAD	5.70	0.07	0.73	0.326	5.38	0.00	0.52	0.369
		HL	5.76	0.06	0.77	0.352	5.76	0.01	0.78	0.397
	(20)	LASSO	4.16	0.00	0.13	0.705	3.89	0.00	0.09	0.666
		SCAD	5.79	0.01	0.80	0.277	5.58	0.00	0.62	0.232
		HL	5.77	0.00	0.80	0.315	5.74	0.00	0.76	0.336
	(20, 30)	LASSO	4.39	0.00	0.19	0.709	3.95	0.00	0.08	0.661
		SCAD	5.79	0.00	0.82	0.289	5.65	0.00	0.70	0.252
		HL	5.81	0.00	0.83	0.323	5.90	0.00	0.91	0.289
	(30, 20)	LASSO	4.16	0.00	0.13	0.793	4.02	0.00	0.11	0.626
		SCAD	5.72	0.00	0.73	0.301	5.60	0.00	0.66	0.273
		HL	5.73	0.00	0.74	0.351	5.82	0.00	0.83	0.299
40%	(10, 20)	LASSO	4.27	0.03	0.15	0.605	3.89	0.01	0.05	0.659
		SCAD	5.76	0.09	0.75	0.364	5.42	0.04	0.54	0.372
		HL	5.71	0.09	0.70	0.380	5.68	0.03	0.73	0.440
	(20)	LASSO	4.32	0.00	0.20	0.669	3.90	0.00	0.06	0.613
		SCAD	5.87	0.00	0.89	0.258	5.65	0.00	0.67	0.207
		HL	5.61	0.00	0.69	0.313	5.76	0.00	0.77	0.296
	(20, 30)	LASSO	4.42	0.00	0.15	0.643	4.21	0.00	0.13	0.685
		SCAD	5.87	0.00	0.89	0.241	5.67	0.00	0.70	0.205
		HL	5.80	0.00	0.82	0.297	5.87	0.00	0.87	0.259
	(30, 20)	LASSO	4.41	0.00	0.20	0.706	4.19	0.00	0.13	0.597
		SCAD	5.85	0.00	0.86	0.275	5.60	0.00	0.65	0.255
		HL	5.78	0.00	0.80	0.312	5.79	0.00	0.80	0.294

*Note:* The true values of dispersion parameters are, respectively, α=0.5, and $\left(\phi_{1},\phi_{2}\right)=\left(3.1,1.5\right)$; Cen.: censoring rate; q: number of clusters; $n_{i}$: cluster size; HL: h‐likelihood penalty function; C: average number of coefficients (of the true zeros, correctly set to zero); IC: average number of the true non‐zeros incorrectly set to zero; PT: probability of choosing the true model; MRME: median of relative model errors.

In addition, we also computed the mean, standard deviation (SD), and SE that are obtained from the sandwich Formula (14) of nonzero coefficients for fixed effects ($\beta_{1}$, $\beta_{2}$) of Types 1 and 2, and dispersion parameters $\left(\phi_{1},\phi_{2},\alpha ,\gamma \right)$. Here, the mean and SD are, respectively, the average and standard deviation of estimated fixed effects (i.e., ${\hat{\beta }}_{1}$ and ${\hat{\beta }}_{2}$) and estimated dispersion parameters $\left({\hat{\phi }}_{1},{\hat{\phi }}_{2},\hat{\alpha },\hat{\gamma }\right)$ under 100 replications when the censoring rate is 20%. The results are summarized in Supporting Information: Table A.1, from which we can see that both SCAD and HL estimates perform well in terms of bias as compared to LASSO estimates. For the estimates of fixed effects, SD is the empirical estimate of the true ${\left\{\mathrm{var}\left({\hat{\beta }}_{k}\right)\right\}}^{1/2}\left(k=1,2\right)$ and SE is the average of 100 estimated standard errors for nonzero‐coefficient estimates ${\left({\hat{\beta }}_{11},{\hat{\beta }}_{14},{\hat{\beta }}_{17}\right)}^{T}$ and ${\left({\hat{\beta }}_{21},{\hat{\beta }}_{24},{\hat{\beta }}_{27}\right)}^{T}$. The SEs are consistently underestimated as compared to SDs for a smaller sample with $\left(q,n_{i}\right)=\left(20,10\right)$. However, the SEs in the SCAD and HL substantially improve in that a discrepancy between SE and SD decreases when q or $n_{i}$ increases to $\left(q,n_{i}\right)=\left(20,20\right),\left(20,30\right)$ or $\left(30,20\right)$, which confirm the simulation results by Ha et al. [16] and Park and Ha [10]. For estimates of dispersion parameters $\left({\hat{\phi }}_{1},{\hat{\phi }}_{2},\hat{\alpha },\hat{\gamma }\right)$, the estimates improve with sample size for three methods (i.e., LASSO, SCAD, and HL).

Supporting Information: Table A.2 summarizes the simulation results under γ=−1. We find that the trends of results from Supporting Information: Table A.2 are similar to those evident in Table 1 under γ=1, and that the SCAD and HL methods still perform well as compared to LASSO.

In addition, we conducted a case with $\beta_{1}={\left(1,0.8,0,0,1,0,0,0.6,0\right)}^{T}$ when γ=1. The simulation results are summarized in Supporting Information: Table A.3. It presents results similar to those of Table 1 and Supporting Information: Table A.2.

### 4.3 An Extended Simulation With More Sparsity

In addition, we conduct simulation studies for an extended case with more sparsity. The simulation design is as follows:

- The sample sizes are $N=\sum_{i=1}^{n}n_{i}=800,1000,1200$, with $\left(n,n_{i}\right)=\left(20,40\right),\left(40,20\right),\left(30,40\right),\left(40,30\right),\left(50,20\right)$, respectively.
- Set the association parameter γ=1.
- The true values of fixed effects (regression coefficients) in Type 1 event are set as follows:

$$\begin{array}{c} \begin{array}{c} \begin{array}{c} \beta_{1}={\left(\beta_{10},\beta_{11},\ldots ,\beta_{1,20}\right)}^{T}=\left(0,0.5,0.5,0.5,0.5,0.5,-0.5,\right.\\ \;{\left.-0.5,-0.5,-0.5,-0.5,0,0,0,0,0,0,0,0,0,0\right)}^{T}. \end{array} \end{array} \end{array}$$

- The remaining simulation settings are the same as those in the simulation of Section 4.1. Here, under the censoring rate of 20%, the proportions of Type 1 [Type 2] events are, respectively, about 40% [40%]; for the 40% censoring, the corresponding proportions are about 30% [30%], respectively.

The simulation results are presented in Supporting Information: Table A.4. The simulation results of variable selection indicate that the SCAD and HL still perform better than the LASSO for Type 1 and Type 2 events, regardless of whether the censoring rate is 20% or 40%. In particular, the HL outperforms the SCAD for both types of events in terms of “C”, “IC”, and “PT” but not for “MRME” when the censoring rate is 20% or 40%.

Based on all the simulation tables and figures, we conclude that HL and SCAD produce generally similar results, which outperform those of LASSO.

## 5 Illustration

In this section, two real data sets on clustered competing risks are utilized to illustrate the proposed penalized variable selection procedure for the cause‐specific joint AFT random‐effect model (1). In this paper, our three variable selection methods (LASSO, SCAD, and HL) essentially identify important variables as those with non‐zero estimates of β. However, we also use statistical significance as an accuracy criterion; a variable is considered significant if the *t*‐test statistic (Estimate/SE) with 1 degree of freedom reaches the 5% significance level. Note that the *t*‐test is approximately equivalent to the z‐test.

### 5.1 Multi‐Center Bladder Cancer Data

We consider a multi‐center bladder cancer data set from a multi‐center clinical trial conducted by the European Organization for Research and Treatment of Cancer (EORTC) [23]. Referring to the bladder cancer data set mentioned in Ha et al. [2], we consider 392 bladder cancer patients from 21 centers, excluding four patients with zero survival times from the original 396 patients. Here, the number of patients in each center varied from 3 to 78, with the mean of 18.9 and the median of 14. The event time of interest, that is, the Type 1 event, is time to the first bladder recurrence; and the competing event, that is, the Type 2 event, is time to the death prior to recurrence. Of the 392 patients, 200 (51.02%) patients had recurrence of bladder cancer, and 81 (20.66%) patients died prior to recurrence. Besides, there were 111 (28.32%) patients still alive without recurrence, that is, they were censored at the date of the last available follow‐up.

There are 9 categorical covariates, which are coded as 12 binary covariates of interest, as follows:

- Chemotherapy (CHEMO, the main covariate): no=0, yes=1,
- Age: 0ifAge≤65years, 1ifAge>65years,
- Sex: male=0,female=1,
- Prior recurrent rate (PRIORREC; primary, ≤1/yr, >1/yr):
$\text{PRIORREC}1=I\left(\text{PRIORREC}\leq 1/\mathrm{yr}\right)$,
$\text{PRIORREC}2=I\left(\text{PRIORREC}>1/\mathrm{yr}\right)$,
- Number of tumors (NOTUM; single, 2−7tumors, ≥8tumors):
$\text{NOTUM}1=I\left(\text{NOTUM}=2-7\;\text{tumors}\right)$,
$\text{NOTUM}2=I\left(\text{NOTUM}\geq 8\;\text{tumors}\right)$,
- Tumor size (TUM3CM): 0 if Tumor size<3cm, 1 if Tumor size≥3cm,
- T category (TLOCC): Ta=0, T1=1,
- Carcinoma in situ (CIS): no=0, yes=1,
- G grade (GLOCAL; G1, G2, G3):
$\text{GLOCAL}1=I\left(\text{GLOCAL}=G2\right)$,
$\text{GLOCAL}2=I\left(\text{GLOCAL}=G3\right)$.

The dataset was also previously analyzed using frailty models [2, 17] and is available in **frailtyHL** R package; the data name is “bladder”.

Table 2 summarizes the estimated coefficients and their corresponding standard errors (SEs) for Type 1 (i.e., bladder cancer recurrence) and Type 2 (i.e., death prior to the recurrence) events under four methods (i.e., no‐penalty, LASSO, SCAD, and HL) using the penalized h‐likelihood procedure for the AFT model (1). Here, for simplicity of variable selection, we do not present the estimates of intercept.

**TABLE 2:** Variable selection results on the cause‐specific AFT model (1) for the bladder cancer data.

Event	Covariate	No‐penalty		LASSO		SCAD		HL	
		Estimate	SE	Estimate	SE	Estimate	SE	Estimate	SE
Type 1	Intercept $\left(x_{0}\right)$	7.227	0.320	6.613	0.230	7.133	0.295	6.884	0.254
	CHEMO $\left(x_{1}\right)$	1.011	0.250	0.906	0.190	1.019	0.251	0.947	0.217
	AGE $\left(x_{2}\right)$	0.287	0.192	0.177	0.105	0	0	0	0
	SEX $\left(x_{3}\right)$	−0.108	0.263	0	0	0	0	0	0
	PRIORREC1 $\left(x_{4}\right)$	−0.261	0.330	0	0	0	0	0	0
	PRIORREC2 $\left(x_{5}\right)$	−0.624	0.260	−0.400	0.151	−0.539	0.247	−0.422	0.172
	NOTUM1 $\left(x_{6}\right)$	−0.776	0.212	−0.473	0.146	−0.772	0.210	−0.614	0.173
	NOTUM2 $\left(x_{7}\right)$	−1.393	0.369	−0.681	0.199	−1.369	0.362	−1.033	0.280
	TUM3CM $\left(x_{8}\right)$	−0.223	0.229	0	0	0	0	0	0
	TLOCC $\left(x_{9}\right)$	−0.288	0.223	−0.156	0.098	0	0	0	0
	CIS $\left(x_{10}\right)$	−0.431	0.390	0	0	0	0	0	0
	GLOCAL1 $\left(x_{11}\right)$	−0.610	0.216	−0.297	0.127	−0.679	0.209	−0.479	0.163
	GLOCAL2 $\left(x_{12}\right)$	−0.942	0.358	−0.422	0.159	−1.188	0.335	−0.830	0.256
Type 2	Intercept $\left(x_{0}\right)$	8.997	0.390	8.284	0.148	8.585	0.188	8.434	0.165
	CHEMO $\left(x_{1}\right)$	−0.508	0.357	0	0	0	0	0	0
	AGE $\left(x_{2}\right)$	−0.912	0.227	−0.535	0.157	−0.851	0.224	−0.691	0.188
	SEX $\left(x_{3}\right)$	0.455	0.290	0.047	0.039	0	0	0	0
	PRIORREC1 $\left(x_{4}\right)$	−0.017	0.363	0	0	0	0	0	0
	PRIORREC2 $\left(x_{5}\right)$	−0.363	0.293	0	0	0	0	0	0
	NOTUM1 $\left(x_{6}\right)$	0.204	0.218	0	0	0	0	0	0
	NOTUM2 $\left(x_{7}\right)$	0.830	0.488	0	0	0	0	0	0
	TUM3CM $\left(x_{8}\right)$	0.193	0.245	0.001	0.001	0	0	0	0
	TLOCC $\left(x_{9}\right)$	−0.057	0.227	0	0	0	0	0	0
	CIS $\left(x_{10}\right)$	−0.471	0.411	0	0	0	0	0	0
	GLOCAL1 $\left(x_{11}\right)$	−0.237	0.223	−0.056	0.053	0	0	0	0
	GLOCAL2 $\left(x_{12}\right)$	0.076	0.420	0	0	0	0	0	0
	${\hat{\phi }}_{1}$	2.715		2.713		2.777		2.726	
	${\hat{\phi }}_{2}$	1.479		1.491		1.611		1.557	
	$\hat{\alpha }$	0.112		0.107		0.113		0.109	
	$\hat{\gamma }$	0.170		0.378		0.297		0.334	
	$\lambda^{*}$	0		0.010		0.096		0.036	

*Note:* The variable selection procedures are based on 4 methods (i.e., no‐penalty, LASSO, SCAD, and HL) for the proposed model (1). (${\hat{\phi }}_{1}$, ${\hat{\phi }}_{2}$, $\hat{\alpha }$, $\hat{\gamma }$): estimates of dispersion parameters ($\phi_{1}$, $\phi_{2}$, α, γ). $\lambda^{*}$: the optimal tuning parameter.

Abbreviations: HL, h‐likelihood penalty function; SE, standard error.

Firstly, the model under no‐penalty is the same as the model by (1). Thus, for the estimations under no‐penalty (i.e., $\sum_{\lambda }=0$ in (11), the results are also the same as model M1's results on table 6 in [6]). Here, the estimated association parameter is $\hat{\gamma }=0.170$, indicating that a positive γ coefficient implies a positive correlation between the two event times of Types 1 and 2. The estimated variance of the random effect is $\hat{\alpha }=0.112$. For the Type 1 event, the effects of six covariates (i.e., CHEMO $\left(x_{1}\right)$, PRIORREC2 $\left(x_{5}\right)$, NOTUM1 $\left(x_{6}\right)$, NOTUM2 $\left(x_{7}\right)$, GLOCAL1 $\left(x_{11}\right)$, GLOCAL2 $\left(x_{12}\right)$) are significant at a 5% significant level among all 12 covariates according to *t*‐values (Estimate/SE). Particularly, the effect of the main covariate, that is, CHEMO $\left(x_{1}\right)$, is the most significant. However, it is not significant for the Type 2 event, and only the effect of AGE $\left(x_{2}\right)$ is significant.

Furthermore, the selection of important variables under three methods (i.e., LASSO, SCAD, and HL) using the proposed penalized h‐likelihood procedure is of interest. The estimates of dispersion parameters $\theta =\left(\phi_{1},\phi_{2},\alpha ,\gamma \right)$ based on the three methods (i.e., LASSO, SCAD, and HL) are, respectively, shown in Table 2, which present somewhat similar results. Obviously, under all three methods, the effect of the main covariate CHEMO is very significant for the Type 1 but not for the Type 2 event. Moreover, for the Type 1 event, LASSO chooses eight covariates (i.e., CHEMO $\left(x_{1}\right)$, AGE $\left(x_{2}\right)$, PRIORREC2 $\left(x_{5}\right)$, NOTUM1 $\left(x_{6}\right)$, NOTUM2 $\left(x_{7}\right)$, TLOCC $\left(x_{9}\right)$, GLOCAL1 $\left(x_{11}\right)$, GLOCAL2 $\left(x_{12}\right)$) among 12 covariates, while both SCAD and HL choose the same 6 covariates (i.e., CHEMO $\left(x_{1}\right)$, PRIORREC2 $\left(x_{5}\right)$, NOTUM1 $\left(x_{6}\right)$, NOTUM2 $\left(x_{7}\right)$, GLOCAL1 $\left(x_{11}\right)$, GLOCAL2 $\left(x_{12}\right)$) of 12 covariates. Note here that the LASSO selects two covariates (i.e., AGE $\left(x_{2}\right)$, TLOCC $\left(x_{9}\right)$) whose effects are not significant at a 5% significant level in terms of *t*‐test under no‐penalty. For the Type 2 event, the LASSO chooses 4 covariates (i.e., AGE $\left(x_{2}\right)$, SEX $\left(x_{3}\right)$, TUM3CM $\left(x_{8}\right)$, GLOCAL1 $\left(x_{11}\right)$), of which the effects of 3 covariates (i.e., SEX $\left(x_{3}\right)$, TUM3CM $\left(x_{8}\right)$, GLOCAL1 $\left(x_{11}\right)$) are not significant under no‐penalty, whereas both SCAD and HL choose the same covariate (i.e., AGE $\left(x_{2}\right)$) among 12 covariates. Besides, the SCAD presents much more similar results of the nonzero estimates to the corresponding significant estimates without penalty (i.e., λ=0), compared to LASSO and HL, meaning that SCAD shrinks the least among the three methods. As shown in Table 2, LASSO appears to overselect, which is consistent with the simulation results (Tables 1 and Supporting Information: Tables A.1–A.3) [10, 16]. As mentioned above, CHEMO is the main covariate and is significantly selected by two methods (SCAD and HL). Its effect is interpreted as follows: the recurrence time (Type 1) in the CHEMO group, according to the SCAD and HL methods, is increased by a factor of $\exp\left(1.021\right)=2.77$ and $\exp\left(0.949\right)=2.58$, respectively, as compared to the placebo group, yielding similar results.

In addition, as shown in Supporting Information: Table A.5, the variable selection results of our penalized AFT model are similar to those of both the penalized cause‐specific frailty model [17] and the penalized sub‐distribution hazard frailty model [3]. The three penalized models consistently select 6 covariates (CHEMO $\left(x_{1}\right)$, PRIORREC2 $\left(x_{5}\right)$, NOTUM1 $\left(x_{6}\right)$, NOTUM2 $\left(x_{7}\right)$, GLOCAL1 $\left(x_{11}\right)$, GLOCAL2 $\left(x_{12}\right)$). From this, we can also see that the variable selection results are, respectively, similar across the three penalty methods, that is, LASSO, SCAD, and HL.

In summary, both SCAD and HL give much simpler models than no‐penalty and LASSO for the multi‐center bladder cancer data set, indicating that the former provides better inferences, interpretation, and prediction than the latter [13].

Moreover, we present the estimated coefficients and their corresponding standard errors (SEs) for two types of events under three methods (LASSO, SCAD, and HL) using the penalized h‐likelihood procedure for the AFT model (1), when the intercept is not penalized, based on the bladder cancer data. The results are summarized in Supporting Information: Table A.6, from which we can see that the three methods, respectively, select almost the same variables as in Table 2 (with penalizing the intercept), except for SEX $\left(x_{3}\right)$ and TUM3CM $\left(x_{8}\right)$ in the Type 2 event. The results indicate that the method without penalizing the intercept does not impact our results much.

The R code of the proposed method for the bladder cancer data is available at: https://github.com/HaoL089/VS_Joint‐AFT_CR.

### 5.2 Multi‐Center BMT Data

Bone marrow transplantation (BMT) is one of the most commonly used treatments for leukaemia patients. A multi‐center study of BMT for leukaemia patients was conducted at four hospitals in Australia and the United States, from March 1, 1984 to June 30, 1989 [4]. As the graft‐versus‐host disease (GVHD) may develop during this period, the recovery after transplantation is quite a complex process [24].

One hundred and thirty seven (137) patients from four hospitals with either acute myelocytic leukaemia (AML) or acute lymphoblastic leukaemia (ALL) were enrolled for the study. The number of patients per hospital varied from 17 to 76, with the mean of 34.25 and the median of 22. Total 82 patients died or relapsed. Here, the event of interest (main event or Type 1 event) is death or relapse, and the competing event (Type 2 event) is GVHD. The details of this study can be found in Copelan et al. [25]. The numbers of Types 1 and 2 events and no event (censoring) are 46 (33.6%), 71 (51.8%), and 20 (14.6%), respectively. Here, 46 was calculated as 82−36=46, where 36 cases had GVHD before death or relapse. The survival data within the same hospital may be correlated due to unobservable shared commonality among patients, thus the cluster effect in the competing risk analysis cannot be ignored [4].

The potential risk factors (covariates) we consider are as follows [4]:

- AML risk group: AML.Low=1, AML.High=2, otherwise=0,
- Donor age in years (D.age),
- Donor sex (D.sex): Male=1, Female=0,
- Donor cytomegalovirus immune status (D.CMV): CMVPositive=1, CMVNegative=0,
- French–American–British classification based on standard morphological criteria for AML patients (FAB): FAB grade 4 or 5 and AML = 1, otherwise = 0,
- The methotrexate used as a Graft‐Versus‐Host‐Prophylactic (MTX): Yes=1, No=0.

Similar to Table 2, the estimates of regression parameters and corresponding SEs based on the four methods for the multi‐center BMT data set are summarized in Table 3. Type 1 event in this data set is death or relapse, whereas the Type 2 event is GVHD. Note that the continuous covariate “D.age” is standardized before variable selection.

**TABLE 3:** Variable selection results on the cause‐specific AFT model (1) for the BMT data.

Event	Covariate	No‐penalty		LASSO		SCAD		HL	
		Estimate	SE	Estimate	SE	Estimate	SE	Estimate	SE
Type 1	Intercept $\left(x_{0}\right)$	6.638	0.976	0	0	0	0	0	0
	AML.Low $\left(x_{1}\right)$	1.218	0.602	0.794	0.246	1.522	0.408	1.244	0.343
	AML.High $\left(x_{2}\right)$	−0.325	0.654	−0.206	0.110	0	0	0	0
	D.age $\left(x_{3}\right)$	−0.136	0.209	−0.047	0.058	0	0	0	0
	D.sex $\left(x_{4}\right)$	−0.027	0.453	0	0	0	0	0	0
	D.CMV $\left(x_{5}\right)$	0.431	0.448	0	0	0	0	0	0
	FAB $\left(x_{6}\right)$	−0.735	0.534	−0.185	0.105	0	0	0	0
	MTX $\left(x_{7}\right)$	−1.267	1.155	0	0	0	0	0	0
Type 2	Intercept $\left(x_{0}\right)$	4.536	0.570	0	0	0	0	0	0
	AML.Low $\left(x_{1}\right)$	1.173	0.481	0.239	0.138	0	0	0	0
	AML.High $\left(x_{2}\right)$	0.851	0.553	0	0	0	0	0	0
	D.age $\left(x_{3}\right)$	−0.516	0.209	−0.313	0.147	0	0	0	0
	D.sex $\left(x_{4}\right)$	0.740	0.377	0.298	0.153	0	0	0	0
	D.CMV $\left(x_{5}\right)$	0.066	0.382	0	0	0	0	0	0
	FAB $\left(x_{6}\right)$	−0.186	0.458	0	0	0	0	0	0
	MTX $\left(x_{7}\right)$	0.313	0.585	0.233	0.118	0	0	0	0
	${\hat{\phi }}_{1}$	3.493		3.502		3.710		3.736	
	${\hat{\phi }}_{2}$	3.589		3.787		3.766		4.339	
	$\hat{\alpha }$	1.063		88.462		69.135		72.197	
	$\hat{\gamma }$	0.384		0.944		0.957		0.958	
	$\lambda^{*}$	0		0.021		0.100		0.087	

*Note:* The variable selection procedures are based on 4 methods (i.e., no‐penalty, LASSO, SCAD, and HL) for the cause‐specific joint AFT model. (${\hat{\phi }}_{1}$, ${\hat{\phi }}_{2}$, $\hat{\alpha }$, $\hat{\gamma }$): estimates of dispersion parameters ($\phi_{1}$, $\phi_{2}$, α, γ). $\lambda^{*}$: the optimal tuning parameter.

Abbreviations: HL, h‐likelihood penalty function; SE, standard error.

First, for the estimated results under no‐penalty, the effect of AML.Low $\left(x_{1}\right)$ is significant for the Type 1 event, whereas the effects of AML.Low $\left(x_{1}\right)$ and D.age $\left(x_{3}\right)$ are significant for the Type 2 event in terms of *t*‐test at a 5% significant level. The estimated association parameter is $\hat{\gamma }=0.384$, indicating a positive dependency between survival times in the two types of events. The estimated variances of random error terms in the two types of events are, respectively, ${\hat{\phi }}_{1}=3.493$ and ${\hat{\phi }}_{2}=3.589$, and the estimated variance of the random effect is $\hat{\alpha }=1.063$.

Second, for the penalized variable selection, we use three methods, that is, LASSO, SCAD, and HL. The optimal values of tuning parameters based on BIC are, respectively, 0.021, 0.100, and 0.087 for LASSO, SCAD, and HL. For Type 1 event, all three methods (i.e., LASSO, SCAD, and HL) select the covariate (i.e., AML.Low $\left(x_{1}\right)$) whose effect is significant. Besides, LASSO selects some covariates (i.e., AML.High $\left(x_{2}\right)$, D.age $\left(x_{3}\right)$, FAB $\left(x_{6}\right)$) whose effects are not significant. For Type 2 event, SCAD and HL select no covariates, whereas LASSO selects D.sex $\left(x_{4}\right)$ and MTX $\left(x_{7}\right)$ whose effects are not significant under no penalty. Thus, the SCAD and HL provide much simpler models than the LASSO.

Similar to Supporting Information: Table A.6, Supporting Information: Table A.7 summarizes the estimated coefficients and their corresponding standard errors (SEs) for two types of events in the BMT data under three methods (i.e., LASSO, SCAD, and HL) using the penalized h‐likelihood procedure for the AFT model (1) when the intercept is not penalized. We can see that the three methods, respectively, select almost the same variables as in Table 3 (with penalizing the intercept), except for MTX $\left(x_{7}\right)$ in the Type 2 event. The results again confirm that the method without penalizing the intercept does not impact our results much.

## 6 Discussion

Penalized variable selection approaches (i.e., LASSO, SCAD, and HL) have been presented for the cause‐specific joint AFT random‐effect model (1) using the penalized h‐likelihood procedure. In this paper, the fixed parameters and random effects for variable selection are estimated using the h‐likelihood method, which circumvents the intractable integration over random effects required in marginal likelihood approaches, resulting in a computationally efficient procedure. In particular, unlike the classical likelihood, which is constructed only for fixed parameters, the h‐likelihood is formulated simultaneously for both fixed parameters and unobserved random effects [2, 13].

Simulation studies showed that the SCAD and HL are more appropriate than the LASSO. In particular, the SCAD overall outperforms HL in a simple design, whereas the HL method overall performs better than the SCAD in a complex design with more sparsity. The proposed method was illustrated with two real data sets, demonstrating that the SCAD and HL perform much better than LASSO.

Developing the penalized AFT competing‐risks models (1) with high‐dimensional covariates $\left(n<<p\right)$ would be an interesting future research. Further extensions of the proposed AFT method are to develop the penalized variable selections under (i) more general multistate models (e.g., illness‐death model) [26, 27], (ii) correlated random effects [4, 15], or (iii) intra‐cluster correlation among event times [28]. In particular, further explanation of case (iii) is necessary, as outlined below. The correlation of $T_{\mathrm{ij}1}$ and $T_{\mathrm{ij}2}$ under (1) is created by the frailty $V_{i}$. On the other hand, the same correlation arises between $T_{\mathrm{ij}1}$ and $T_{\mathrm{ik}1}$ for j<k (intra‐cluster correlation), as well as between $T_{\mathrm{ij}2}$ and $T_{\mathrm{ik}2}$ for j<k. Some studies model competing risks dependence using a copula, while employing a frailty to account for intra‐cluster dependence [28].

## Conflicts of Interest

The authors declare no conflicts of interest.

## Supporting information


**Data S1:** pst70084‐sup‐0001‐Supinfo.pdf.

## Data Availability

The data that support the findings of this study are openly available in frailtyHL at https://cran.r‐project.org/web/packages/frailtyHL/index.html.
