# Supplementary material for: Penalized Variable Selection for Joint AFT Random‐Effect Model With Clustered Competing‐Risks Data
Source: Pharm Stat. 2026 Mar 15;25(2):e70084. doi: 10.1002/pst.70084 (PMC12989290; doi:10.1002/pst.70084)
Supplement: Supplementary file 1 — Data S1: pst70084‐sup‐0001‐Supinfo.pdf. [file PST-25-0-s001.pdf]

SUPPLEMENTARY MATERIAL:

“Penalized Variable Selection for Joint AFT Random-effect Model with Clustered Competing-Risks Data"

By Lin Hao and Il Do Ha

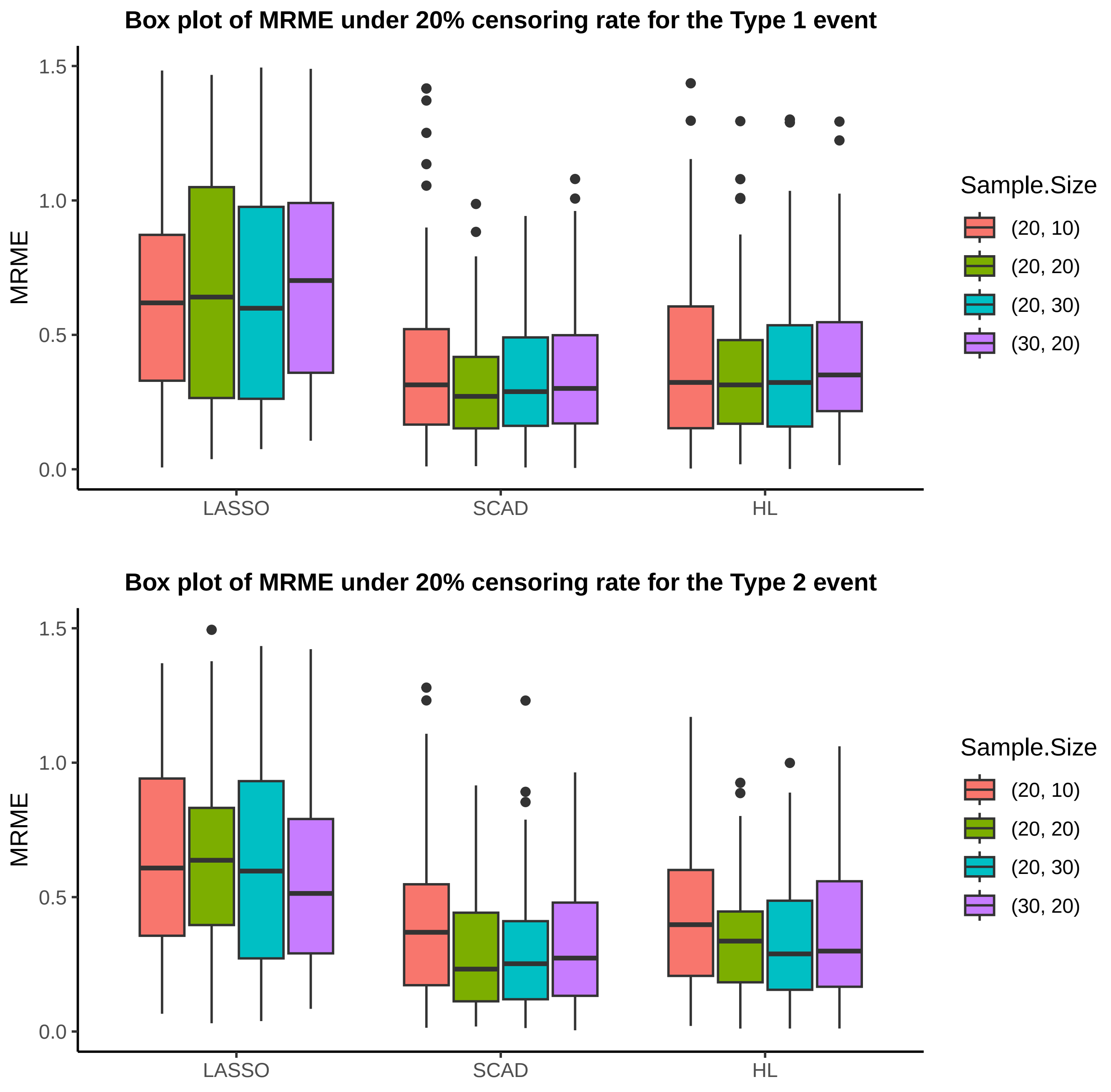

FIGURE A.1 Box plots for “MRME” of three methods under both types of events when the censoring rate is 20%.

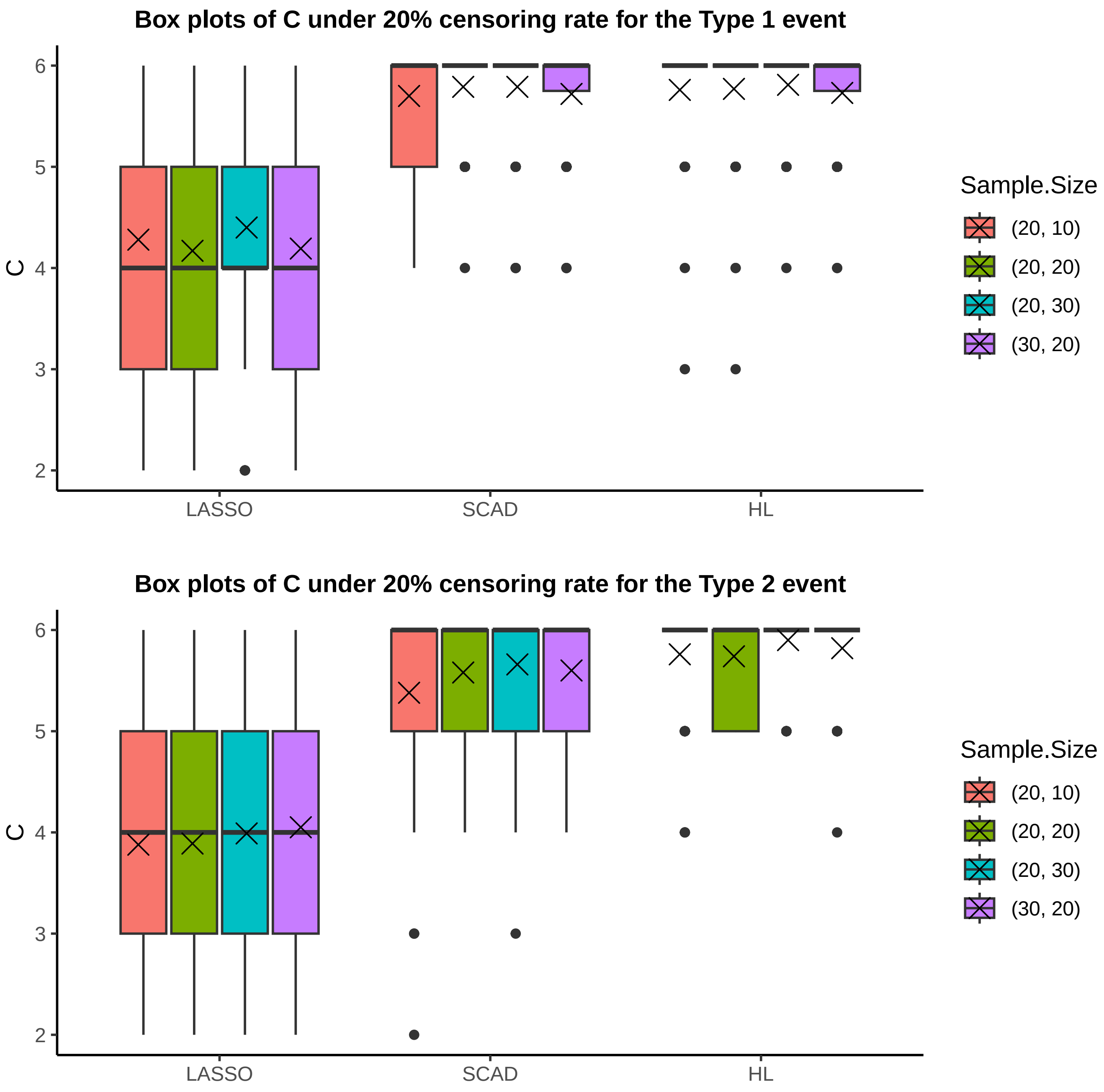

FIGURE A.2 Box plots for “C” of three methods under both types of events when the censoring rate is 20%.

□

TABLE A.1 Simulation results for nonzero regression coefficients and dispersion parameters $(\phi_1, \phi_2, \alpha, \gamma)$ under the cause-specific AFT model (1).

(q, n_i)	Method	Type1						Type2						$\hat{\phi}_1$		$\hat{\phi}_2$		$\hat{\alpha}$		$\hat{\gamma}$	
		$\hat{\beta}_{11}$		$\hat{\beta}_{14}$		$\hat{\beta}_{17}$		$\hat{\beta}_{11}$		$\hat{\beta}_{14}$		$\hat{\beta}_{17}$									
		Mean	SD (SE)	Mean	SD (SE)	Mean	SD (SE)	Mean	SD (SE)	Mean	SD (SE)	Mean	SD (SE)	Mean	SD	Mean	SD	Mean	SD	Mean	SD
	TRUE	0.8		1		0.6		-0.8		-1		-0.6		3.1		1.5		0.5		1	
	LASSO	0.612	0.169 (0.117)	0.811	0.168 (0.127)	0.392	0.148 (0.101)	-0.696	0.145 (0.100)	-0.838	0.134 (0.105)	-0.469	0.129 (0.093)	3.065	0.491	1.524	0.262	0.532	0.284	1.081	0.436
(20, 10)	SCAD	0.810	0.165 (0.155)	1.016	0.166 (0.158)	0.557	0.203 (0.136)	-0.826	0.140 (0.117)	-0.983	0.121 (0.121)	-0.587	0.135 (0.113)	3.181	0.486	1.595	0.276	0.530	0.291	1.076	0.447
	HL	0.718	0.171 (0.137)	0.932	0.169 (0.144)	0.483	0.176 (0.119)	-0.771	0.136 (0.109)	-0.928	0.123 (0.113)	-0.535	0.127 (0.103)	3.122	0.479	1.564	0.260	0.519	0.282	1.069	0.413
	LASSO	0.672	0.114 (0.089)	0.861	0.118 (0.094)	0.480	0.125 (0.082)	-0.709	0.085 (0.073)	-0.903	0.093 (0.077)	-0.500	0.081 (0.069)	2.952	0.314	1.503	0.167	0.527	0.229	1.045	0.240
(20, 20)	SCAD	0.813	0.104 (0.105)	1.013	0.110 (0.108)	0.611	0.135 (0.102)	-0.800	0.083 (0.081)	-1.012	0.087 (0.084)	-0.589	0.082 (0.070)	3.065	0.318	1.565	0.175	0.528	0.229	1.068	0.279
	HL	0.769	0.106 (0.100)	0.965	0.114 (0.103)	0.564	0.128 (0.095)	-0.770	0.084 (0.078)	-0.977	0.087 (0.081)	-0.561	0.081 (0.076)	3.016	0.312	1.540	0.172	0.524	0.228	1.060	0.260
	LASSO	0.701	0.087 (0.074)	0.880	0.099 (0.077)	0.483	0.080 (0.070)	-0.711	0.070 (0.060)	-0.911	0.070 (0.063)	-0.518	0.060 (0.057)	2.935	0.262	1.487	0.140	0.524	0.229	1.014	0.208
(20, 30)	SCAD	0.815	0.090 (0.085)	1.005	0.099 (0.087)	0.597	0.083 (0.084)	-0.789	0.069 (0.065)	-1.001	0.071 (0.067)	-0.594	0.059 (0.063)	3.035	0.266	1.540	0.146	0.522	0.236	1.031	0.223
	HL	0.785	0.090 (0.082)	0.972	0.101 (0.084)	0.565	0.083 (0.080)	-0.770	0.069 (0.063)	-0.982	0.072 (0.066)	-0.575	0.058 (0.062)	3.000	0.267	1.525	0.143	0.517	0.232	1.028	0.220
	LASSO	0.705	0.083 (0.074)	0.869	0.093 (0.078)	0.487	0.099 (0.070)	-0.727	0.062 (0.059)	-0.914	0.068 (0.063)	-0.518	0.070 (0.057)	2.950	0.288	1.452	0.137	0.508	0.207	1.040	0.208
(30, 20)	SCAD	0.816	0.083 (0.086)	0.992	0.095 (0.088)	0.600	0.098 (0.085)	-0.801	0.063 (0.065)	-1.001	0.066 (0.067)	-0.588	0.070 (0.063)	3.040	0.301	1.501	0.141	0.497	0.212	1.062	0.231
	HL	0.787	0.083 (0.083)	0.961	0.096 (0.086)	0.569	0.101 (0.080)	-0.782	0.063 (0.063)	-0.981	0.067 (0.066)	-0.572	0.068 (0.061)	3.008	0.293	1.487	0.140	0.497	0.209	1.057	0.223

Censoring rate: 20%;

q: No. of clusters; n_i: cluster size;

SD: standard deviation for estimates over 100 replications;

SE: average of estimated standard errors over 100 replications.

TABLE A.2 Simulation results using 100 replications for variable selection in the cause-specific AFT model (1) under $\gamma = -1$.

Cen.	(q, n_i)	Method	Type1				Type2			
			C	IC	PT	MRME	C	IC	PT	MRME
20%	(20, 10)	LASSO	4.26	0.00	0.14	0.677	3.87	0.00	0.07	0.633
		SCAD	5.70	0.03	0.73	0.330	5.48	0.00	0.64	0.276
		HL	5.76	0.07	0.76	0.319	5.75	0.01	0.78	0.364
	(20, 20)	LASSO	4.33	0.00	0.14	0.617	3.77	0.00	0.04	0.539
		SCAD	5.82	0.00	0.83	0.272	5.59	0.00	0.64	0.236
		HL	5.77	0.00	0.78	0.321	5.76	0.00	0.77	0.312
	(20, 30)	LASSO	4.35	0.00	0.14	0.569	3.74	0.00	0.05	0.564
		SCAD	5.89	0.00	0.89	0.272	5.54	0.00	0.63	0.208
		HL	5.79	0.00	0.79	0.295	5.79	0.00	0.81	0.288
	(30, 20)	LASSO	4.23	0.00	0.14	0.744	3.81	0.00	0.10	0.575
		SCAD	5.89	0.00	0.90	0.262	5.64	0.00	0.71	0.235
		HL	5.75	0.00	0.79	0.303	5.78	0.00	0.79	0.330
40%	(20, 10)	LASSO	4.44	0.00	0.15	0.694	4.16	0.00	0.11	0.599
		SCAD	5.87	0.08	0.82	0.284	5.65	0.01	0.67	0.287
		HL	5.80	0.06	0.80	0.350	5.78	0.01	0.79	0.308
	(20, 20)	LASSO	4.46	0.00	0.19	0.721	4.03	0.00	0.09	0.591
		SCAD	5.85	0.00	0.86	0.303	5.67	0.00	0.68	0.291
		HL	5.81	0.00	0.83	0.362	5.80	0.00	0.81	0.355
	(20, 30)	LASSO	4.45	0.00	0.18	0.669	3.87	0.00	0.10	0.557
		SCAD	5.89	0.00	0.91	0.238	5.74	0.00	0.76	0.228
		HL	5.80	0.00	0.82	0.384	5.75	0.00	0.77	0.314
	(30, 20)	LASSO	4.37	0.00	0.16	0.615	4.04	0.00	0.06	0.543
		SCAD	5.94	0.00	0.94	0.217	5.71	0.00	0.75	0.256
		HL	5.76	0.00	0.76	0.295	5.90	0.00	0.91	0.323

TABLE A.3 Simulation results using 100 replications for variable selection in the cause-specific AFT model (1) under $\beta_1 = (1, 0.8, 0, 0, 1, 0, 0, 0.6, 0)^T$ with $\beta_2 = -\beta_1$ and $\gamma = 1$.

Cen.	(q, n_i)	Method	Type1				Type2			
			C	IC	PT	MRME	C	IC	PT	MRME
20%	(20, 10)	LASSO	3.23	0.30	0.09	2.537	2.43	0.00	0.02	0.855
		SCAD	4.62	0.58	0.40	0.955	4.42	0.00	0.58	0.705
		HL	4.66	0.62	0.36	1.201	4.76	0.00	0.80	0.678
	(20, 20)	LASSO	3.08	0.02	0.10	2.561	2.23	0.00	0.01	0.890
		SCAD	4.81	0.07	0.78	0.738	4.38	0.01	0.59	0.682
		HL	4.73	0.05	0.74	0.512	4.76	0.01	0.79	0.741
	(20, 30)	LASSO	3.09	0.00	0.09	3.081	2.24	0.00	0.01	0.888
		SCAD	4.91	0.08	0.84	0.708	4.64	0.00	0.68	0.629
		HL	4.77	0.04	0.74	0.645	4.83	0.00	0.85	0.745
	(30, 20)	LASSO	3.13	0.01	0.10	4.389	2.40	0.00	0.01	0.847
		SCAD	4.89	0.04	0.87	0.649	4.51	0.00	0.65	0.658
		HL	4.82	0.03	0.80	0.978	4.78	0.00	0.79	0.697
40%	(20, 10)	LASSO	3.45	0.60	0.07	2.389	2.77	0.00	0.06	0.892
		SCAD	4.77	1.06	0.23	1.200	4.45	0.00	0.56	0.785
		HL	4.72	1.12	0.17	1.799	4.81	0.00	0.81	0.815
	(20, 20)	LASSO	3.21	0.10	0.07	2.980	2.25	0.00	0.02	0.913
		SCAD	4.87	0.24	0.68	0.778	4.47	0.00	0.59	0.810
		HL	4.81	0.22	0.65	1.170	4.71	0.00	0.75	0.817
	(20, 30)	LASSO	3.07	0.06	0.06	2.604	2.23	0.00	0.02	0.917
		SCAD	4.92	0.17	0.77	0.739	4.68	0.01	0.70	0.673
		HL	4.84	0.14	0.71	0.750	4.81	0.00	0.82	0.763
	(30, 20)	LASSO	3.17	0.04	0.10	4.695	2.36	0.00	0.00	0.835
		SCAD	4.85	0.14	0.75	0.716	4.62	0.00	0.71	0.675
		HL	4.73	0.15	0.66	1.353	4.82	0.00	0.83	0.713

The true values of dispersion parameters are $\alpha = 0.5$ and $(\phi_1, \phi_2) = (3.1, 1.5)$.
Cen.: censoring rate; q: number of clusters; n_i: cluster size; HL: h-likelihood penalty function.
C: average number of coefficients (of the true zeros, correctly set to zero).
IC: average number of the true non-zeros incorrectly set to zero.
PT: probability of choosing the true model.
MRME: median of relative model errors.

TABLE A.4 Simulation results using 100 replications for variable selection in the cause-specific AFT model (1) under an extended case with more sparsity.

Cen.	(q, n_i)	Method	Type1				Type2			
			C	IC	PT	MRME	C	IC	PT	MRME
20%	(20, 40)	LASSO	9.03	0.00	0.15	0.981	7.80	0.00	0.02	1.023
		SCAD	10.59	0.02	0.63	0.425	10.07	0.00	0.41	0.424
		HL	10.67	0.01	0.70	0.552	10.65	0.00	0.72	0.588
	(40, 20)	LASSO	9.08	0.00	0.12	0.997	7.73	0.00	0.02	0.962
		SCAD	10.73	0.02	0.74	0.392	10.16	0.00	0.41	0.440
		HL	10.76	0.00	0.77	0.523	10.65	0.00	0.68	0.590
	(30, 40)	LASSO	8.37	0.00	0.13	0.878	7.17	0.00	0.02	0.752
		SCAD	10.65	0.00	0.73	0.413	10.19	0.00	0.46	0.430
		HL	10.73	0.00	0.76	0.440	10.62	0.00	0.68	0.467
	(40, 30)	LASSO	8.29	0.00	0.12	0.786	7.15	0.00	0.03	0.773
		SCAD	10.67	0.00	0.71	0.444	10.28	0.00	0.47	0.470
		HL	10.64	0.00	0.69	0.591	10.69	0.00	0.73	0.505
	(20, 50)	LASSO	9.13	0.00	0.18	1.002	7.88	0.00	0.06	0.925
		SCAD	10.73	0.02	0.75	0.396	10.24	0.00	0.47	0.407
		HL	10.80	0.00	0.82	0.529	10.64	0.00	0.68	0.457
40%	(20, 40)	LASSO	9.15	0.01	0.20	0.990	8.13	0.00	0.06	1.002
		SCAD	10.70	0.15	0.62	0.434	10.36	0.02	0.47	0.405
		HL	10.74	0.04	0.76	0.523	10.66	0.00	0.71	0.561
	(40, 20)	LASSO	9.21	0.00	0.27	0.876	7.99	0.00	0.06	0.878
		SCAD	10.75	0.12	0.70	0.404	10.21	0.01	0.46	0.445
		HL	10.79	0.03	0.79	0.504	10.73	0.00	0.77	0.503
	(30, 40)	LASSO	7.66	0.00	0.05	0.628	6.16	0.00	0.00	0.567
		SCAD	10.83	0.00	0.84	0.344	10.33	0.00	0.53	0.383
		HL	10.68	0.00	0.71	0.478	10.64	0.00	0.69	0.408
	(40, 30)	LASSO	7.50	0.00	0.03	0.485	6.52	0.00	0.01	0.723
		SCAD	10.68	0.00	0.74	0.351	10.43	0.00	0.54	0.403
		HL	10.64	0.00	0.70	0.387	10.73	0.00	0.74	0.521
	(20, 50)	LASSO	8.37	0.00	0.13	0.641	7.52	0.00	0.05	0.714
		SCAD	10.68	0.02	0.72	0.391	10.35	0.00	0.53	0.383
		HL	10.75	0.00	0.78	0.462	10.69	0.00	0.72	0.393

The true values of dispersion parameters are, respectively, $\alpha = 0.5$, and $(\phi_1, \phi_2) = (3.1, 1.5)$.
Cen.: censoring rate; q: number of clusters; n_i: cluster size; HL: h-likelihood penalty function.
C: average number of coefficients (of the true zeros, correctly set to zero).
IC: average number of the true non-zeros incorrectly set to zero.
PT: probability of choosing the true model.
MRME: median of relative model errors.

TABLE A.5 Estimated regression coefficients (standard errors) in three types of penalized models for the Type 1 event (recurrence) in the bladder cancer data.

Covariate	LASSO			SCAD			HL		
	CS AFT	Sub Frailty	CS Fraitly	CS AFT	Sub Frailty	CS Fraitly	CS AFT	Sub Frailty	CS Fraitly
CHEMO (x_1)	0.906 (0.190)	-0.666 (0.166)	-0.598 (0.142)	1.019 (0.251)	-0.929 (0.182)	-0.870 (0.182)	0.947 (0.217)	-0.785 (0.174)	-0.696 (0.158)
AGE (x_2)	0.177 (0.105)	-0.214 (0.120)	-0.131 (0.079)	0 (0)	0 (0)	0 (0)	0 (0)	-0.218 (0.119)	0 (0)
SEX (x_3)	0 (0)	0 (0)	0 (0)	0 (0)	0 (0)	0 (0)	0 (0)	0 (0)	0 (0)
PRIORREC1 (x_4)	0 (0)	0 (0)	0 (0)	0 (0)	0 (0)	0 (0)	0 (0)	0 (0)	0 (0)
PRIORREC2 (x_5)	-0.400 (0.151)	0.327 (0.149)	0.336 (0.119)	-0.539 (0.247)	0.395 (0.180)	0.426 (0.178)	-0.422 (0.172)	0.294 (0.150)	0.337 (0.127)
NOTUM1 (x_6)	-0.473 (0.146)	0.494 (0.139)	0.455 (0.118)	-0.772 (0.210)	0.688 (0.164)	0.671 (0.163)	-0.614 (0.173)	0.593 (0.150)	0.514 (0.131)
NOTUM2 (x_7)	-0.681 (0.199)	0.816 (0.229)	0.693 (0.171)	-1.369 (0.362)	1.293 (0.272)	1.213 (0.269)	-1.033 (0.280)	1.051 (0.249)	0.863 (0.209)
TUM3CM (x_8)	0 (0)	0.060 (0.094)	0.002 (0.002)	0 (0)	0 (0)	0 (0)	0 (0)	0 (0)	0 (0)
TLOCC (x_9)	-0.156 (0.098)	0.127 (0.115)	0.183 (0.091)	0 (0)	0 (0)	0 (0)	0 (0)	0 (0)	0 (0)
CIS (x_{10})	0 (0)	0 (0)	0 (0)	0 (0)	0 (0)	0 (0)	0 (0)	0 (0)	0 (0)
GLOCAL1 (x_{11})	-0.297 (0.127)	0.250 (0.126)	0.269 (0.103)	-0.679 (0.209)	0.491 (0.159)	0.540 (0.159)	-0.479 (0.163)	0.384 (0.137)	0.356 (0.119)
GLOCAL2 (x_{12})	-0.422 (0.159)	0.347 (0.189)	0.276 (0.111)	-1.188 (0.335)	0.910 (0.250)	0.914 (0.249)	-0.830 (0.256)	0.610 (0.222)	0.552 (0.178)

CS AFT: the penalized cause-specific AFT model whose results are from Table 2.
Sub Frailty: the penalized sub-distribution hazard frailty model by Ha et al. (2014b).
CS Fraitly: the penalized cause-specific frailty model by Rakhmawati et al. (2021).

TABLE A.6 Variable selection results on the cause-specific AFT model (1), without penalizing the intercept, for the bladder cancer data.

Event	Covariate	LASSO		SCAD		HL	
		Estimate	SE	Estimate	SE	Estimate	SE
Type 1	Intercept (x_0)	7.004	0.228	7.133	0.295	7.002	0.252
	CHEMO (x_1)	0.681	0.177	1.019	0.251	0.846	0.211
	AGE (x_2)	0.110	0.078	0	0	0	0
	SEX (x_3)	0	0	0	0	0	0
	PRIORREC1 (x_4)	0	0	0	0	0	0
	PRIORREC2 (x_5)	-0.447	0.152	-0.539	0.247	-0.440	0.170
	NOTUM1 (x_6)	-0.523	0.147	-0.772	0.210	-0.622	0.170
	NOTUM2 (x_7)	-0.689	0.193	-1.369	0.362	-1.002	0.270
	TUM3CM (x_8)	0	0	0	0	0	0
	TLOCC (x_9)	-0.221	0.111	0	0	0	0
	CIS (x_{10})	0	0	0	0	0	0
	GLOCAL1 (x_{11})	-0.339	0.130	-0.679	0.209	-0.492	0.161
	GLOCAL2 (x_{12})	-0.383	0.143	-1.188	0.335	-0.800	0.245
Type 2	Intercept (x_0)	8.462	0.155	8.585	0.188	8.414	0.161
	CHEMO (x_1)	0	0	0	0	0	0
	AGE (x_2)	-0.631	0.164	-0.851	0.224	-0.663	0.182
	SEX (x_3)	0	0	0	0	0	0
	PRIORREC1 (x_4)	0	0	0	0	0	0
	PRIORREC2 (x_5)	0	0	0	0	0	0
	NOTUM1 (x_6)	0	0	0	0	0	0
	NOTUM2 (x_7)	0	0	0	0	0	0
	TUM3CM (x_8)	0	0	0	0	0	0
	TLOCC (x_9)	0	0	0	0	0	0
	CIS (x_{10})	0	0	0	0	0	0
	GLOCAL1 (x_{11})	-0.101	0.073	0	0	0	0
	GLOCAL2 (x_{12})	0	0	0	0	0	0
	$\hat{\phi}_1$	2.749		2.777		2.741	
	$\hat{\phi}_2$	1.562		1.611		1.550	
	$\hat{\alpha}$	0.084		0.113		0.102	
	$\hat{\gamma}$	0.309		0.297		0.330	
	λ^*	0.011		0.100		0.023	

TABLE A.7 Variable selection results on the cause-specific AFT model (1), without penalizing the intercept, for the BMT data.

Event	Covariate	LASSO		SCAD		HL	
		Estimate	SE	Estimate	SE	Estimate	SE
Type 1	Intercept (x_0)	6.544	0.877	5.982	0.747	5.817	0.682
	AML.Low (x_1)	0.739	0.242	1.498	0.465	1.222	0.376
	AML.High (x_2)	-0.225	0.107	0	0	0	0
	D.age (x_3)	-0.012	0.018	0	0	0	0
	D.sex (x_4)	0	0	0	0	0	0
	D.CMV (x_5)	0	0	0	0	0	0
	FAB (x_6)	-0.256	0.133	0	0	0	0
	MTX (x_7)	0	0	0	0	0	0
Type 2	Intercept (x_0)	6.625	0.597	6.784	0.544	5.774	0.224
	AML.Low (x_1)	0.232	0.136	0	0	0	0
	AML.High (x_2)	0	0	0	0	0	0
	D.age (x_3)	-0.039	0.019	0	0	0	0
	D.sex (x_4)	0.273	0.150	0	0	0	0
	D.CMV (x_5)	0	0	0	0	0	0
	FAB (x_6)	0	0	0	0	0	0
	MTX (x_7)	0	0	0	0	0	0
	$\hat{\phi}_1$	3.644		3.761		3.781	
	$\hat{\phi}_2$	3.781		3.972		4.130	
	$\hat{\alpha}$	1.692		1.473		1.560	
	$\hat{\gamma}$	0.151		0.182		0.180	
	λ^*	0.021		0.100		0.092	
